# Good guardian, bad parent: tradeoffs between territory defense and parental care in Darwin's finches

**DOI:** 10.1093/beheco/araf109

**Published:** 2025-09-24

**Authors:** Andrew C Katsis, Lauren K Common, Çağlar Akçay, Sonia Kleindorfer

**Affiliations:** Konrad Lorenz Research Center for Behavior and Cognition, University of Vienna, Fischerau 13, 4645 Grünau im Almtal, Austria; Department of Behavioral and Cognitive Biology, University of Vienna, Universitätsring 1, 1010 Vienna, Austria; Konrad Lorenz Research Center for Behavior and Cognition, University of Vienna, Fischerau 13, 4645 Grünau im Almtal, Austria; Department of Behavioral and Cognitive Biology, University of Vienna, Universitätsring 1, 1010 Vienna, Austria; School of Life Sciences, Anglia Ruskin University, East Road, Cambridge, Cambridgeshire CB1 1PT, United Kingdom; Konrad Lorenz Research Center for Behavior and Cognition, University of Vienna, Fischerau 13, 4645 Grünau im Almtal, Austria; Department of Behavioral and Cognitive Biology, University of Vienna, Universitätsring 1, 1010 Vienna, Austria; College of Science and Engineering, Flinders University, Sturt Road, Bedford Park, South Australia 5042, Australia

**Keywords:** aggressiveness, Darwin's finches, parental care, personality, territory defense

## Abstract

Although defending a territory may benefit individuals by allowing them to retain important resources, the time and energy costs associated with territory defense may lead territory owners to neglect other reproductively important behaviors. In this study, we assessed the potential tradeoff between territory defense and parental care in 4 Darwin's finch species on Floreana Island, Galápagos. Using song playback, we simulated territory intrusions to measure male aggressiveness across multiple stages of the breeding cycle (unpaired, paired, incubating, and chick feeding). To quantify parental care at each nest, we conducted 1-h observations to record the frequency of male food deliveries and the duration of female incubation and brooding. Male aggressiveness toward a perceived intruder did not change across the breeding cycle and was highly repeatable (adjusted *R* = 0.597), although responses became less vocal across the breeding cycle. A male's aggressiveness did not predict his frequency of food deliveries during incubation or chick feeding, although females paired with more aggressive males spent significantly less time incubating. This finding provides weak evidence for a tradeoff between territory defense and parental care, although the behavioral mechanisms mediating this relationship remain uncertain. Finally, males with aggressive and nonaggressive behavioral phenotypes did not differ in their hatching success, although additional work is needed to assess other measures of individual fitness.

## Teaser

Although territory holders retain priority access to important resources, defending a territory can divert time and energy away from other important behaviors, such as parental care. In this study, we assessed whether male Darwin’s finches that were more aggressive during territory defense faced such a tradeoff during the breeding season. Contrary to our predictions, more aggressive males did not deliver less food to the nest, although their females spent significantly less time incubating.

## Introduction

Many animal species occupy and defend a home territory (Maher and Lott 2000; Hinsch and Komdeur 2017). By maintaining a patch of habitat to which they have priority access, territory owners may retain important resources, such as food or breeding partners (Cain et al. 2015; Krieg and Getty 2020). However, maintaining a territory can also be very costly, and for such behavior to be adaptive its benefits must outweigh its costs (Hinsch and Komdeur 2010; Ord 2021). Territory maintenance involves activities such as “posting” (vocally signaling at the territory boundaries) or scent marking to advertise territory boundaries, as well as more direct interactions such as ritualized aggression or physical altercations between territory owners and intruders (Ord 2021). The costs most commonly associated with territory defense include patrolling effort (time spent guarding against intruders), ownership advertisement (signals broadcasting ownership of the territory), physical defense (stress, energy, and injury costs associated with contesting intruders), risk of predation (increased conspicuousness while defending a territory), and time away from other activities (diversion of time away from nonterritorial behaviors) (Ord 2021).

When facing a perceived intruder, not all territory owners are equally aggressive: how aggressively territory owners defend against a perceived intruder has been shown to have consistent individual variation, as indicated by high levels of individual repeatability over time (eg Akçay et al. 2014; Thys et al. 2017; Salazar et al. 2021). Why individuals should differ in aggressive territory defense—and how this variation is maintained in populations—remains an open question (Maynard Smith and Harper 1988). One potential explanation is that the relative costs and benefits of aggressiveness differ between individuals, leading to distinct optimal strategies when choosing how much to invest in territory defense (Marler et al. 1995; Hyman et al. 2004). Indeed, the costs and benefits associated with territory defense are likely influenced by a combination of intrinsic factors (eg age or experience of the territory owner) and extrinsic factors (eg territory quality or intrusion pressure) (Hyman et al. 2004; Szász et al. 2019a).

When individuals within a population face differing tradeoffs, they may adopt distinct territory defense strategies with similar fitness payoffs (Maynard Smith and Price 1973). Although aggressively defending a territory would allow some individuals to retain access to important resources, the associated time and energy costs could lead them to neglect other important behaviors, such as foraging or mating (Ord 2021). During the breeding season in particular, there may be a direct tradeoff between territory maintenance and parental care. For example, meerkat (*Suricata suricatta*) parents reduced their offspring food provisioning when intruders were present in the territory (Mares et al. 2012). Similarly, blue tits (*Cyanistes caeruleus*) that were more aggressive during territory defense fed their offspring less frequently (Mutzel et al. 2013) and female tree swallows (*Tachycineta bicolor*) that were more aggressive raised slower-growing chicks (Rosvall 2011). This apparent tradeoff between territory defense and parental care suggests the potential for 2 behavioral strategies with equal fitness payoff: more aggressive territory owners maximize their access to resources while less aggressiveness territory owners instead invest more strongly in parental care. According to the “challenge hypothesis,” this tradeoff may be partly mediated by endocrinal mechanisms, whereby males that experience frequent territorial interactions have increased testosterone secretion, which, in turn, reduces their parental care effort (Wingfield et al. 1990, 2020; but see Goymann et al. 2019). Importantly, the hypothesized tradeoff between territory defense and parental care is not universal in the literature (Stiver and Alonzo 2009), with several studies finding no correlation between these variables (Szász et al. 2019b; Lane et al. 2023).

In this study, we assessed the proposed tradeoff between male territory defense and parental care in Darwin's finches. Male Darwin's finches are highly territorial during the breeding season, defending a small area around their nest before and after pairing (Ratcliffe and Grant 1983, 1985; Akçay et al. 2024). Males are also heavily involved in nest provisioning, bringing food for their partner during incubation and to their offspring posthatching (Kleindorfer et al. 2021). Here, we experimentally measured territory defense behavior in the field by broadcasting conspecific song to simulate a territory intruder. Our experiments spanned multiple breeding stages of the same individuals (before pairing, after pairing, incubation, and chick feeding) to assess how territory defense changed across the breeding cycle. To quantify parental care at these same nests, we then conducted 1-h nest observations during incubation and chick feeding to record (1) male food deliveries during incubation, (2) female incubation time, (3) male food deliveries during chick feeding, and (4) female brooding time. First, we predicted that among-individual differences in male territory defense behavior would be repeatable over time. Second, we expected a tradeoff between aggressiveness and parental care, with more aggressive males making fewer food deliveries to the nest, and a consequent decrease in female incubation and brooding time. Finally, to assess any potential fitness consequences of territory defense behavior, we tested whether more aggressive males during the incubation period were more or less likely to hatch offspring.

## Materials and methods

### Study site and species

We conducted our study from January to March 2024 on Floreana Island in the Galápagos archipelago, at 2 highland (humid zone) sites and 1 lowland (dry zone) site. The 2 highland sites, located at Asilo de la Paz (1°18′46″S 90°27′16″W) and the base of Cerro Pajas (1°17′46″S 90°27′06″W), consisted largely of remnant *Scalesia pedunculata* forest. The 1 lowland (dry zone) site comprised dry scrubland dominated by palo santo trees (*Bursera graveolens*) in and around the township of Puerto Velasco Ibarra (1°16′28″S 90°29′13″W). Our study included 4 of the 5 Darwin's finch species considered extant on the island: the small ground finch (*Geospiza fuliginosa*), common cactus finch (*Geospiza scandens*), small tree finch (*Camarhynchus parvulus*), and medium tree finch (*Camarhynchus pauper*). Small ground finches and small tree finches were present at all 3 sites, medium tree finches only breed at the 2 highland sites, and cactus finches only at the lowlands site. We did not identify any nests belonging to the island's fifth extant species, the medium ground finch (*Geospiza fortis*).

During the breeding season, unpaired male Darwin's finches build a display nest inside their territory and sing at the nest until chosen by a female (Grant 1986). After mating, the female lays 3 to 4 eggs and incubates them for a period of ∼12 d, during which the male delivers food to her (Grant 1986). After hatching, the female broods the chicks and both parents deliver food (Kleindorfer et al. 2021), until fledging occurs 13 to 16 d posthatch (Grant 1986). At the time of this study (February to March 2025), the major causes of Darwin's finch nest failure were egg and nestling predation by the introduced smooth-billed ani (*Crotophaga ani*) and chick parasitism by the larvae of invasive avian vampire flies (*Philornis downsi*) (Kleindorfer and Dudaniec 2016). Historically, Darwin's finch eggs and nestlings were also susceptible to predation by black rats (*Rattus rattus*), house mice (*Mus musculus*), and feral cats (*Felis catus*) (O’Connor et al. 2010), but these predators were seldom observed on Floreana Island immediately following an island-wide eradication campaign that began in October 2023 (Kleindorfer et al. pers. obs.). Another common nest predator, the native short-eared owl (*Asio flammeus galapagoensis*), was only rarely observed on the island during the study period, with a subset of the population taken into captivity to mitigate the risk of secondary poisoning during the mammal eradication campaign (Island Conservation 2013). The predator eradication program is ongoing, with phase 2 planned for 2027, and we note that some short-eared owls and mammalian predators were present on Floreana Island by early 2025 (Kleindorfer et al. pers. obs.); however, these predators did not noticeably contribute to nest mortality during the timeline of this study.

### Nest monitoring

We searched for Darwin's finch nests at our 3 study sites and monitored the breeding activity of male finches whose territory defense behavior we had previously measured. Upon discovering an active display nest, we observed the male for ∼20 min to determine if he was paired or unpaired. We then revisited the nest approximately every 3 d to confirm the male's current status. If the male became paired, we continued to regularly monitor the breeding pair to determine the success of their nesting attempt. To establish the breeding stage (incubation or chick feeding), we either used a borescope to visually confirm the presence of eggs or nestlings or observed the parents' incubation or provisioning behavior with binoculars, following a standardized protocol (Kleindorfer et al. 2021). When observing parental behavior, the observer was seated on the ground, ∼10 m from the nest, with binoculars focused on the nest entrance. As Darwin's finches do not noticeably alter their behavior in the presence of a human observer within 5 m (Goodale and Podos 2010), this observer distance was unlikely to disrupt parental behavior. All nest observations took place in the morning, between 06:00 and 12:00 Galapagos time (GALT), to minimize time-of-day effects.

Monitoring ended when the outcome of the nesting attempt was known (ie nest abandoned or taken over, offspring died, or offspring fledged). For each nest that reached the incubation stage, we used its hatching success (eggs did not hatch, eggs hatched) as a measure of reproductive success. We did not consider rates of fledging success in this study, due to coinciding experiments that would have confounded this fitness measure. Specifically, many Darwin's finch nests during the study period experienced a reduction in avian vampire fly parasite loads, either due to manual insecticide spraying by the research team or because Darwin's finches collected insecticide-treated nest lining material from feather dispensers placed every 50 m in the study area (Common et al. in review).

### Territory defense trials

Male Darwin's finches defend their territories from conspecific intruders by displaying aggressively and chasing intruders from the area (Ratcliffe and Grant 1983, 1985). To quantify this territory defense behavior, we used conspecific song playback to simulate male intruders in a territory, using similar protocols to Colombelli-Négrel et al. (2023) and Katsis et al. (2024). We specifically targeted the male nest owner, as female Darwin's finches rarely respond to song playback (Ratcliffe and Grant 1985).

Our playback stimuli were constructed from Darwin's finch songs recorded on Floreana Island in 2023. These songs were recorded as uncompressed 16-bit broadcast wave files (.wav) using a Sennheiser ME67 or MKE600 directional microphone (Sennheiser electronic GmbH & Co., USA) connected to either a Marantz PMD 660 Solid State recorder (Sound United, LLC, USA) or a Zoom H5 recorder (Zoom North America, USA). We created 25 unique playback tracks (8 small ground finch, 8 small tree finch, 7 medium tree finch, 2 cactus finch), each lasting 3 min (1 min of song playback, 1 min of silence, 1 min of song playback). Each 1-min song playback period contained 6 repetitions of the same male song type, simulating a territory intrusion by a single unfamiliar conspecific. We used a high-pass filter to remove sounds <1 kHz and saved the playback tracks as uncompressed 16-bit wave files, which were then transferred to an Apple iPod (Apple Inc., USA). Songs were broadcast in the field using a Sony XB12 Extra Bass Portable Bluetooth Speaker (Sony Australia Limited), with a frequency response of 20 Hz to 20 kHz.

Upon entering a Darwin's finch territory, we placed the speaker in the branches of a shrub or tree ∼5 m from the nest, at a height of 1 to 1.5 m. Playback tracks were randomly assigned for each trial, with the provision that each male received a conspecific song. We never started playback until the male was observed within 20 m of the speaker; in cases where the male was unbanded, we waited until he returned to the nest or interacted with the female to ensure we were observing the nest owner. After playback began, an observer (A.C.K. or S.K.) narrated the male's response into a digital audio recorder. For each trial, we quantified 6 response variables during the playback period: time (in seconds) within 5 m of the speaker, time (in seconds) within 1 m of the speaker, minimum distance (in meters) from the speaker, number of flights, number of crosses (ie flights that crossed the speaker), and number of vocalizations (defined in Table 1). For a subset of trials (*N* = 24 of 168 trials), we also recorded the male's behavior during a 1-min baseline period prior to the beginning of playback.

**Table 1.** Operational definitions for behaviors recorded during simulated territory intrusions and nest observations in Darwin's finches.

Variable	Definition
Simulated territory intrusions	
Time within 5 m^a^	Duration (seconds) within 5 m of the speaker
Time within 1 m^a^	Duration (seconds) within 1 m of the speaker
Minimum distance^a^	Minimum distance (meters) from the speaker
Flights^a^	Number of flights
Crosses^a^	Number of flights that crossed over the speaker
Total vocalizations	Number of vocalizations (songs, buzzes, or whistles) produced
Nest observations	
Female incubation time	Duration (minutes) spent inside the nest by the female during the incubation stage
Female brooding time	Duration (minutes) spent inside the nest by the female during the chick feeding stage
Male food deliveries	Number of food deliveries by the male, either to the female (during either breeding stage) or to the chicks (during the chick feeding stage)

^a^Variable contributed to *PC_TerritoryDefense*, our measure of territory defense behavior.

We conducted all playback experiments in the morning, between 06:00 and 12:00 GALT to correspond with the peak of song activity. Across 168 playback trials, we tested males at 98 nests (37 small ground finch, 28 medium tree finch, 27 small tree finch, 6 cactus finch). Of these, 2 small ground finch nests belonged to the same color-banded male across consecutive breeding attempts. An additional 2 nests were tested but excluded from our dataset due to uncertainty about the species identification. Territory defense trials were conducted across 4 stages of the breeding cycle: unpaired (male was unpaired and defending a display nest), paired (male was paired with a female), incubation (female was incubating eggs), and chick feeding (male and female were feeding chicks).

### Parental care observations

During incubation and chick feeding, we conducted 1-h parental care observations at Darwin's finch nests between 06:00 and 12:00 GALT, following long-term standardized protocols for this study population (eg Kleindorfer et al. 2021). Observers were positioned ∼10 m from the nest with binoculars focused on the nest entrance. During the incubation stage, we recorded: (1) number of male food deliveries to the incubating female at or near the nest entrance, and (2) total female incubation time (minutes). During the chick feeding stage, we recorded: (3) number of male food deliveries to the chicks or female; and (4) total female brooding time (minutes). In other songbird species, 1-h observations were sufficient to provide an accurate measure of daily provisioning rate (in blue tits, García-Navas and Sanz 2012; in great tits, *Parus major*, Pagani-Núñez and Senar 2013; in tree swallows, Lendvai et al. 2015), although this has not been validated in Darwin's finches.

In total, we conducted 113 parental care observations at 59 nests (24 small ground finch, 18 small tree finch, 15 medium tree finch, 2 cactus finch). By breeding stage, we conducted 76 observations at 53 nests during the incubation period (mean ± SE observations per nest = 1.43 ± 0.07, range 1 to 3) and 37 observations at 28 nests during the chick feeding period (1.32 ± 0.12 observations per nest, range 1 to 3). Repeat observations were not always possible, either because the nest was discovered late in the breeding stage or due to premature nest failure. For each observation, we estimated the within-stage timing of the breeding attempt based on our timeline of monitoring records. Given our monitoring schedule, this estimate was usually considered precise to within 3 d. Using this estimate, we then broadly assigned each protocol to the early (days 1 to 7) or late (days 8 to 14) stage of incubation or chick feeding. A subset of observations (*N* = 11 observations at 10 nests) could not be confidently assigned to a within-stage category due to inadequate monitoring.

### Statistical analysis

All statistical analyses were performed in R v. 4.2.3 (R Core Team 2023). Principal component analyses were performed on the correlation matrix using the *princomp* function in the package “stats” v. 4.2.3. Linear mixed models and generalized linear models used the “lmer” and “glm” functions, respectively, in lme4 package v. 1.1.32 (Bates et al. 2015).

#### Creation of PC_TerritoryDefense variable

Using principal component analysis, we reduced our territory defense variables to 1 uncorrelated principal component named PC_TerritoryDefense, which had eigenvalue 2.91 and explained 58.3% of variability (Table 2). Initially, this analysis included all 6 response variables. Following the recommendations of Björklund (2019), we used the R package “PCAtest” (Camargo 2022) to calculate the ψ and ϕ statistics of the principal component analysis, the distinctness of the first principal component (eigenvalue), and the significance of each variable's eigenvectors on this principal component. We used bootstrap resampling of the observed data (nboot = 1,000) to calculate confidence intervals (CIs) and random permutations (nperm = 1,000) to build null distributions within each variable (Camargo 2022). Significant ψ and ϕ statistics (ψ = 4.86, *P* < 0.001; ϕ = 0.40, *P* < 0.001) indicated that our territory defense data had a nonrandom correlational structure and, hence, that the analysis was biologically meaningful (Björklund 2019). PC1 was significantly distinct (*P* < 0.001) from the other principal components; therefore, we only retained the first principal component (renamed PC_TerritoryDefense). Before further analysis, we omitted 1 response variable (total vocalizations) from the analysis because it did not load significantly on PC_TerritoryDefense (Björklund 2019); total vocalizations was instead analyzed as a separate response variable.

**Table 2.** Eigenvectors for the first principal component extracted from 5 response variables recorded during simulated territory intrusions.

Parameter	PC1 eigenvector
PC_TerritoryDefense	
Time within 5 m	0.46
Time within 1 m	0.38
Minimum distance	−0.52
Flights	0.46
Crosses	0.41
PC_MeanTerritoryDefense	
Time within 5 m	0.46
Time within 1 m	0.39
Minimum distance	−0.51
Flights	0.46
Crosses	0.41

PC_TerritoryDefense (2.91 eigenvalue, 58.3% of variance explained) was extracted from the per-trial responses and PC_MeanTerritoryDefense (3.03 eigenvalue, 60.7% of variance explained) was extracted from individual means per breeding stage.

#### Comparison of baseline and playback periods

For a subset of our playback trials (*N* = 24), we observed male behavior during a 1-min period prior to the beginning of playback. To confirm that subjects were responding to the simulated territory intrusion, we used paired *t*-tests to compare all 6 variables (time within 5 m, time within 1 m, minimum distance, flights, crosses, and total vocalizations) between the baseline period and the first minute of song playback.

#### Factors affecting territory defense

To assess which factors predict territory defense response, we performed 2 linear mixed models with PC_TerritoryDefense and total vocalizations, respectively, as the response variable. Both models included trial number (0 to 6), species (small ground finch, common cactus finch, small tree finch, medium tree finch), breeding stage (unpaired, paired, incubation, chick feeding), and study site (Puerto Velasco Ibarra, Cerro Pajas, Asilo de la Paz) as fixed effects, and male identity as a random effect. To normalize the residuals in the second model, total vocalizations was square-root-transformed. To explicitly compare the total number of vocalizations between each breeding stage, we conducted post hoc pairwise comparisons using the *pair* function in the package “emmeans” v. 1.8.5 (Lenth 2022), with *P*-values Holm-adjusted to account for multiple testing.

#### Repeatability of territory defense

Using the 2 linear mixed models described above, we calculated the adjusted repeatability (adjusted *R*) of both PC_TerritoryDefense and total vocalizations. This is the proportion of total variance in each response variable that is explained by differences between individuals, after accounting for trial number, species, breeding stage, and study site (Nakagawa and Schielzeth 2010). To calculate repeatability, we used the package rptR v. 0.9.22 (Stoffel et al. 2017), which relies on mixed-effects models fitted using lme4. The statistical significance of repeatability was tested using likelihood ratio tests against a null hypothesis that *R* = 0, while 95% CIs were estimated by parametric bootstrapping (1,000 iterations). The dataset for calculating the repeatability of territory defense included subjects tested only once, since they contribute to the estimate of total variance.

#### Relationship between territory defense and parental care

Because some nest owners were tested twice at the same breeding stage, we first derived a single territory defense score per individual per breeding stage (PC_MeanTerritoryDefense). To do so, we calculated each individual's mean value per breeding stage for 5 playback response variables (time within 5 m, time within 1 m, minimum distance, flights, and crosses). We then used principal component analysis to reduce these 5 variables to a single principal component with eigenvalue 3.03 and explaining 60.7% of variability. This principal component analysis had similar eigenvectors to those previously calculated using the per-trial data (Table 2).

We used linear mixed models to test the relationship between male territory defense and parental care. For nest observations conducted during incubation, we used 2 models with male food deliveries and female incubation time, respectively, as response variables. These models included PC_MeanTerritoryDefense (during the incubation stage), male species (small ground finch, small tree finch, medium tree finch), study site (Puerto Velasco Ibarra, Cerro Pajas, Asilo de la Paz), and within-stage timing (early or late incubation) as fixed effects, and nest identity as a random effect to control for repeated observations at the same nest. For nest observations conducted during chick feeding, we used 2 linear mixed models with male food deliveries and female brooding time, respectively, as response variables. These models included PC_MeanTerritoryDefense (during the chick feeding stage), male species, study site, and within-stage timing as fixed effects, and nest identity as a random effect. All 4 models initially included an additional interaction term (species × territory defense) to allow for species-specific tradeoffs between territory defense and parental care, but these interactions were all nonsignificant (*P* > 0.05) and subsequently removed from the models.

#### Relationship between territory defense and hatching success

We restricted our analysis to nests with a known hatching outcome whose male was tested during the incubation stage. This subset included 37 nests (17 small ground finch, 15 small tree finch, 5 medium tree finch). To assess whether male territory defense predicted hatching success, we used a binomial generalized linear model with hatching success (no, yes) as the response variable and PC_MeanTerritoryDefense, male species, and study site as fixed effects.

### Ethics

All fieldwork procedures were conducted with approval from the Austrian Federal Ministry for Science and Research (EU Standard, equivalent to the Animal Ethics Board) with Animal Experiment License Number 66.006/0026-WF/V/3b/2014. Permission to conduct this research was granted by the Galápagos National Park Directorate (PC-87-23).

## Results

### Comparison of baseline and playback periods

For a subset of playback trials, we recorded 1 min of baseline behavior prior to song playback (summary data in Table 3; *N* = 24 trials at 24 nests, including 11 small ground finches, 8 small tree finches, 4 medium tree finches, 1 cactus finch). During the first minute of playback, time within 5 m (*t* = 3.73, *P* = 0.001), flights (*t* = 7.11, *P* < 0.001), and crosses (*t* = 5.78, *P* < 0.001) significantly increased compared with the baseline period, with a near-significant increase in time within 1 m (*t* = 1.98, *P* = 0.060). The minimum distance significantly decreased between the baseline period and the first minute of playback (*t* = −5.86, *P* < 0.001). The total number of vocalizations did not differ between these 2 periods (*t* = −1.24, *P* = 0.227).

**Table 3.** Summary data comparing 6 behavioral variables (time within 5 m, time within 1 m, minimum distance, flights, crosses, vocalizations) in male Darwin's finches before and during a simulated territory intrusion.

Variable	Baseline			Minute 1			Minute 2			Minute 3		
	Mean	SE	Range	Mean	SE	Range	Mean	SE	Range	Mean	SE	Range
Subset of playback trials with baseline data (*N* = 24)												
Time within 5 m (s)	11.2	4.3	0–60	33.8	3.9	0–60	31.4	5.6	0–60	37.7	4.8	0–60
Time within 1 m (s)	0.0	0.0	0	3.0	1.5	0–33	3.3	1.8	0–37	6.8	2.4	0–36
Minimum distance (m)	7.8	0.5	4.0–10.0	3.7	0.6	0.5–10.0	4.0	0.6	1.0–10.0	3.2	0.6	0.5–10.0
Flights	1.4	0.3	0–5	6.5	0.8	1–16	3.0	0.5	0–11	5.8	0.7	0–12
Crosses	0.0	0.0	0–1	2.2	0.4	0–5	0.3	0.1	0–2	2.0	0.4	0–6
Total vocalizations	5.0	1.0	0–20	3.8	0.6	0–9	5.5	0.8	0–14	4.1	0.8	0–13
Full dataset of playback trials (*N* = 168)												
Time within 5 m (s)	…	…	…	35.0	1.5	0–60	32.4	1.9	0–60	35.9	1.6	0–60
Time within 1 m (s)	…	…	…	2.8	0.5	0–36	2.2	0.5	0–51	4.5	0.8	0–47
Minimum distance (m)	…	…	…	3.3	0.2	0–10.0	4.1	0.2	0.3–10.0	3.5	0.2	0.2–10.0
Flights	…	…	…	6.6	0.3	0–16	3.4	0.2	0–11	6.0	0.3	0–13
Crosses	…	…	…	2.1	0.1	0–7	0.3	0.0	0–3	2.1	0.1	0–7
Total vocalizations	…	…	…	2.2	0.3	0–14	3.7	0.3	0–16	2.6	0.2	0–13

Each trial comprised 1 min of song playback (minute 1), 1 min of silence (minute 2), and 1 min of song playback (minute 3). For a subset of trials, we also recorded male behavior during a 1-min baseline period prior to song playback.

### Factors affecting territory defense

**Fig. 1.**
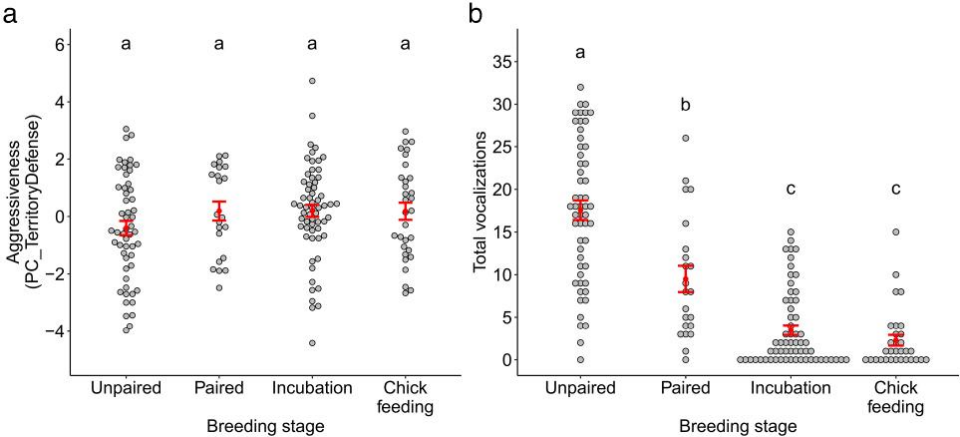
Territory defense in response to a simulated territory intruder across 4 breeding stages (unpaired, paired, incubation, chick feeding) in male Darwin's finches. (a) Higher PC_TerritoryDefense values indicate that the bird spent more time within 5 and 1 m of the speaker, approached more closely to the speaker, and performed more flights and crosses. (b) The number of vocalizations produced during the playback period, including songs, whistles, and (in tree finches) buzzes. Different letters indicate statistically significant differences in response, based on post hoc pairwise comparisons.

**Fig. 2.**
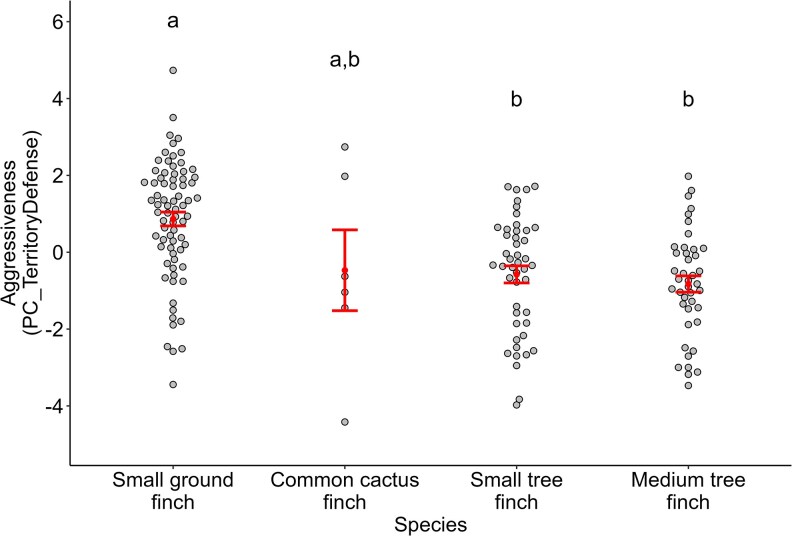
Territory defense in response to a simulated territory intruder across 4 species of Darwin's finch (small ground finch, common cactus finch, small tree finch, medium tree finch). Higher PC_TerritoryDefense values indicate that the bird spent more time within 5 and 1 m of the speaker, approached more closely to the speaker, and performed more flights and crosses. Different letters indicate statistically significant differences in response between species.

We conducted 168 playback trials at 98 nests (37 small ground finch, 28 medium tree finch, 27 small tree finch, 6 cactus finch). Summary data for the 6 territory defense variables are provided in Table 3. Territory defense behavior (PC_TerritoryDefense) did not differ between sites or breeding stages (Fig. 1; Table 4), and small ground finches responded more strongly to playback than did small tree finches or medium tree finches (Fig. 2). Total vocalizations did not differ between sites or species, but did significantly differ between breeding stages (Table 4). Specifically, the number of vocalizations decreased between the unpaired and paired stages and decreased again between the paired and incubation stages (Fig. 1; Table 5).

**Table 4.** Output from linear mixed models testing which factors influence territory defense in response to a simulated territory intrusion, with either PC_TerritoryDefense or total vocalizations as the response variable.

Variable	Estimate	Error	*t*	*P*
PC_TerritoryDefense				
Intercept	0.61	0.53	1.15	0.251
Trial number	0.09	0.12	0.73	0.470
Species (CF)	−1.17	0.81	−1.45	0.150
Species (STF)	−1.86	0.39	−4.72	**<0.001**
Species (MTF)	−2.11	0.42	−5.08	**<0.001**
Breeding stage (paired)	−0.09	0.38	−0.25	0.803
Breeding stage (incubation)	0.09	0.32	0.28	0.778
Breeding stage (feeding)	−0.41	0.42	−0.97	0.332
Study site (Cerro Pajas)	0.45	0.56	0.81	0.422
Study site (Asilo de la Paz)	0.53	0.58	0.90	0.369
Total vocalizations				
Intercept	4.22	0.42	10.12	**<0.001**
Trial number	0.05	0.10	0.52	0.602
Species (CF)	−0.44	0.63	−0.70	0.487
Species (STF)	−0.13	0.31	−0.41	0.681
Species (MTF)	−0.22	0.32	−0.68	0.501
Breeding stage (paired)	−1.53	0.31	−4.94	**<0.001**
Breeding stage (incubation)	−3.01	0.26	−11.71	**<0.001**
Breeding stage (feeding)	−3.22	0.34	−9.58	**<0.001**
Study site (Cerro Pajas)	−0.31	0.43	−0.71	0.478
Study site (Asilo de la Paz)	0.22	0.45	0.50	0.621

Both models included trial number (0 to 6), species (small ground finch, common cactus finch, small tree finch, medium tree finch), breeding stage (unpaired, paired, incubation, chick feeding), and study site (Puerto Velasco Ibarra, Cerro Pajas, Asilo de la Paz) as fixed effects, and male identity as a random effect. Statistically significant (<0.05) values are marked in bold.

Species abbreviations: SGF, small ground finch; CF, common cactus finch; STF, small tree finch; MTF, medium tree finch. For species, “SGF” is the reference category. For breeding stage, “unpaired” is the reference category. For site, “Puerto Velasco Ibarra” is the reference category.

**Table 5.** Post hoc pairwise comparisons comparing the total vocalizations produced during a simulated territory intrusion between 4 stages of the breeding cycle: unpaired, paired, incubation, and chick feeding.

Comparison	Estimate	SE	*t*	*P*-value
Unpaired–paired	1.53	0.31	4.89	**<0.001**
Unpaired–incubation	3.01	0.26	11.61	**<0.001**
Unpaired–chick feeding	3.22	0.34	9.48	**<0.001**
Paired–incubation	1.49	0.27	5.58	**<0.001**
Paired–chick feeding	1.69	0.34	4.92	**<0.001**
Incubation–chick feeding	0.21	0.26	0.80	0.424

Statistically significant (<0.05) values are marked in bold.

### Repeatability of territory defense

Territory defense behavior (PC_TerritoryDefense) during the playback experiment was highly repeatable (adjusted *R* = 0.597, SE = 0.078, 95% CI = 0.471 to 0.761, *P* < 0.001). Similarly, the total number of vocalizations produced during playback trials was highly repeatable (adjusted *R* = 0.514, SE = 0.083, 95% CI = 0.377 to 0.709, *P* < 0.001).

### Relationship between territory defense and parental care

**Fig. 3.**
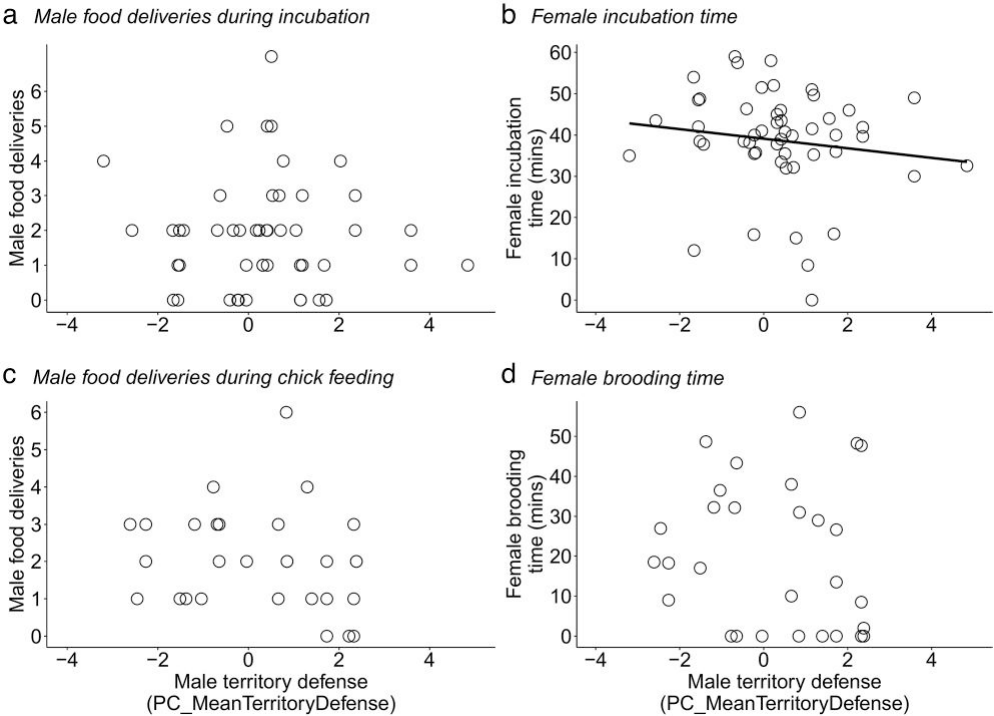
Scatterplots showing the relationship between territory defense and parental care in Darwin's finch nests. Male territory defense: (a) did not predict the number of male food deliveries during incubation; (b) was negatively associated with female incubation time; (c) did not predict the number of male food deliveries during chick feeding; and (d) did not predict female brooding time. Higher PC_MeanTerritoryDefense scores indicate more aggressive responses to a simulated territory intrusion.

During the incubation stage, we conducted 51 nest observations at 38 nests (15 small ground finch, 13 small tree finch, 10 medium tree finch) where we had reliable data on within-stage timing and had also conducted territory defense trials. The number of male food deliveries was not predicted by his territory defense behavior (*t* = 0.47, *P* = 0.641; Fig. 3; Table 6). Male species, study site, and within-stage timing also did not influence the number of male food deliveries (Table 6). Female incubation time was negatively associated with male territory defense: that is, females with more aggressive partners spent less time on the nest (*t* = −2.18, *P* = 0.035; Fig. 3; Table 6). In addition, females incubated for longer during the second half of the incubation stage (Table 6). Female incubation time differed between study sites but not between species (Table 6).

**Table 6.** Output from linear mixed models testing the association between territory defense and parental care (male food deliveries, female incubation/brooding time) during the incubation and chick feeding stages.

Variable	Estimate	Error	*t*	*P*-value
Male food deliveries during incubation				
Intercept	1.15	0.98	1.17	0.250
PC_MeanTerritoryDefense	0.09	0.19	0.47	0.641
Male species (STF)	1.30	0.67	1.95	0.059
Male species (MTF)	1.20	0.74	1.61	0.117
Study site (Cerro Pajas)	0.11	1.01	0.11	0.915
Study site (Asilo de la Paz)	−0.21	1.02	−0.21	0.836
Within-stage timing (late)	0.08	0.32	0.26	0.801
Female incubation time				
Intercept	23.80	6.88	3.46	**0.001**
PC_MeanTerritoryDefense	−2.61	1.20	−2.18	**0.035**
Male species (STF)	−6.22	4.11	−1.52	0.137
Male species (MTF)	−1.18	4.45	−0.26	0.793
Sudy site (Cerro Pajas)	17.91	6.81	2.63	**0.012**
Study site (Asilo de la Paz)	12.44	6.86	1.81	0.077
Within-stage timing (late)	8.00	3.21	2.49	**0.017**
Male food deliveries during chick feeding				
Intercept	1.67	1.10	1.51	0.171
PC_MeanTerritoryDefense	−0.15	0.34	−0.45	0.663
Male species (STF)	−0.40	1.12	−0.36	0.725
Male species (MTF)	0.33	1.31	0.25	0.808
Study site (Cerro Pajas)	0.51	1.37	0.37	0.720
Study site (Asilo de la Paz)	0.54	1.46	0.37	0.720
Within-stage timing (late)	0.06	0.81	0.07	0.946
Female brooding time				
Intercept	28.75	12.66	2.27	**0.041**
PC_MeanTerritoryDefense	3.60	3.93	0.92	0.371
Male species (STF)	13.17	13.02	1.01	0.325
Male species (MTF)	15.36	15.32	1.00	0.328
Study site (Cerro Pajas)	−9.02	15.73	−0.57	0.575
Study site (Asilo de la Paz)	−9.93	16.76	−0.59	0.563
Within-stage timing (late)	−30.17	9.72	−3.11	**0.006**

All models included male territory defense (PC_MeanTerritoryDefense), male species, study site, and within-stage timing as fixed effects, and nest identity as a random effect. Higher PC_MeanTerritoryDefense values indicate more aggressive territory defense responses. Statistically significant (<0.05) values are marked in bold.

Species abbreviations: SGF, small ground finch; STF, small tree finch; MTF, medium tree finch. For species, “SGF” is the reference category. For within-stage timing, “early” is the reference category. For site, “Puerto Velasco Ibarra” is the reference category.

During the chick feeding stage, we conducted 29 nest observations at 20 nests (12 small ground finch, 4 small tree finch, 4 medium tree finch) where we had reliable data on within-stage timing and had also conducted territory defense trials. Neither the number of male food deliveries (*t*  *=* −0.45, *P* = 0.663) nor female brooding time (*t* = 0.92, *P* = 0.371) were predicted by the male's territory defense behavior (Fig. 3; Table 6). Male species and site did not influence either variable, although females brooded less in the late chick feeding period compared with the early chick feeding period (Table 6).

### Relationship between territory defense and hatching success

**Fig. 4.**
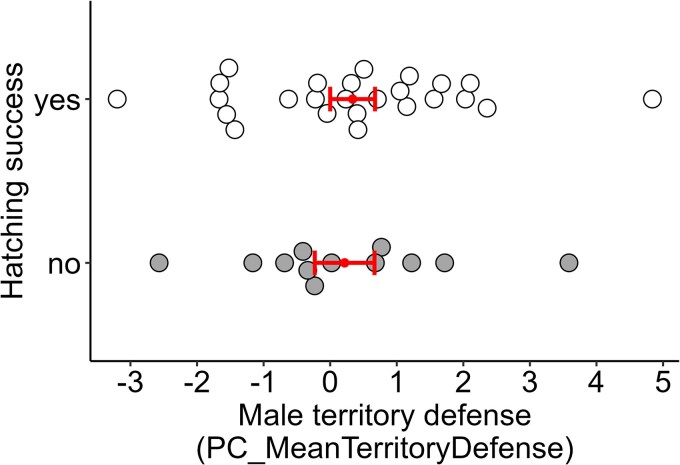
Dotplot showing no significant relationship between male territory defense during the incubation period and hatching success in Darwin's finch nests. Small circles and bars indicate the mean ± SE territory defense score per outcome, with raw data overlaid. Higher PC_MeanTerritoryDefense scores indicate more aggressive responses to a simulated territory intrusion.

We collected territory defense and hatching success data for 37 nests (17 small ground finch, 15 small tree finch, 5 medium tree finch). Male territory defense during the incubation stage did not predict hatching success at his nest (*z* = −0.02, *P* = 0.984; Fig. 4; Table 7) nor did hatching success differ between species or study sites (Table 7).

**Table 7.** Output from a generalized linear model with binomial error distribution testing which factors influence hatching success in Darwin's finches.

Variable	Estimate	Error	*z*	*P*-value
Intercept	18.03	2,200.06	0.01	0.993
PC_MeanTerritoryDefense	−0.01	0.31	−0.02	0.984
Species (STF)	−1.14	0.96	−1.19	0.235
Species (MTF)	0.29	1.51	0.19	0.849
Study site (Cerro Pajas)	−17.26	2,200.06	−0.01	0.994
Study site (Asilo de la Paz)	−16.69	2,200.06	−0.01	0.994

The model contained hatching success (eggs did not hatch, eggs hatched) as the response variable, and male territory defense (PC_MeanTerritoryDefense), male species (small ground finch, small tree finch, medium tree finch), and study site (Puerto Velasco Ibarra, Cerro Pajas, Asilo de la Paz) as fixed effects.

Species abbreviations: SGF, small ground finch; STF, small tree finch; MTF, medium tree finch. For species, “SGF” is the reference category. For site, “Puerto Velasco Ibarra” is the reference category.

## Discussion

Aggressively defending a territory can help individuals retain important resources, but may detract from other important behaviors, particularly during the breeding season (Ord 2021). In this study, we experimentally measured the consistency of territory defense behavior in male Darwin's finches and assessed potential tradeoffs with parental care. Among-individual differences in male territory defense behavior were highly repeatable across the breeding cycle, providing a robust measure of individual aggressiveness in these species. Contrary to predictions, we found no evidence that aggressive males invested less in nest provisioning, with no apparent relationship between a male's territory defense score and his number of food deliveries. However, females that were paired with aggressive males spent less time incubating their eggs. This result partly aligns with the hypothesized tradeoff between male territory defense and parental care, although, without any differences in male-to-female food provisioning, the mechanisms mediating this relationship are unclear. Finally, we found no association between male aggressiveness during the incubation stage and hatching success, which suggests that both nonaggressive and aggressive strategies achieve equal fitness during this stage of the breeding cycle. For logistical reasons, we did not analyze any posthatching measures of breeding success, although it is possible that male territory defense behavior indirectly influences factors such as offspring parasite load, growth rate, or fledging success.

The strength of males' territory defense response remained similar across the 4 stages of the breeding cycle (unpaired, paired, incubation, and chick feeding). This suggests that males do not moderate their aggressive response to intruders after egg laying, despite an increase in parental responsibilities during the incubation and chick feeding stages. The exception to this finding was that males became substantially less vocal across the breeding cycle, with successive decreases in vocal response after pairing and again after egg laying. This shift in vocal behavior may represent a tradeoff between the costs and benefits of singing. In Darwin's finches, male vocalizations serve the dual purposes of territory defense and mate attraction (Grant and Grant 1996; Akçay et al. 2024). Partnered males may have reduced incentive to sing, leading to a decrease in vocal behavior. Similar decreases in vocality have been reported in Eurasian chaffinches (*Fringilla coelebs*), in which singing activity decreased immediately after pair formation (Hanski and Laurila 1993). During incubation, males reduce their vocal rates even further—in many cases, to zero—which could be a strategy to minimize predation risk if frequent song is more likely to draw the attention of nest predators (Kleindorfer et al. 2016). This reduction in vocal response could be driven by endocrinal changes that occur within each male across the breeding cycle: for example, in spotted antbirds (*Hylophylax naevioides*), experimentally blocking the effects of testosterone reduced the number of vocalizations produced during staged male–male encounters (Hau et al. 2000).

Territory defense behavior during a simulated territory intrusion was highly repeatable, making it an ideal in situ measure of aggressiveness in Darwin’s finches. Previous studies in songbirds, including great tits (Thys et al. 2017; Hardman and Dalesman 2018; Naguib et al. 2022), blue tits (Salazar et al. 2021; van Iersel et al. 2023), and song sparrows (*Melospiza melodia*; Hyman et al. 2004; Akçay et al. 2014) support that aggressiveness during territory defense is highly repeatable and represents a consistent personality trait. Because individuals remained in the same location across all trials, these high repeatability estimates may be partly influenced by the characteristics of each territory (Niemelä and Dingemanse 2017). For example, territory owners may react more aggressively toward intruders—regardless of personality—if they occupy territories with greater resource value or that experience higher intrusion rates (eg in little bustards, *Tetrax tetrax*; Morales et al. 2014). Niemelä and Dingemanse (2017) propose overcoming this confound by translocating individuals between territories for repeated testing, although this is not feasible in our study system, as translocated male finches would likely return to their original location. Nevertheless, longer-term testing of individuals across multiple seasons and breeding attempts would help disentangle any environmental influences on territory defense. In addition, we previously found that individuals' territory defense behavior correlates with their aggressiveness toward a mirror during temporary captivity, suggesting that these among-individual differences are maintained even when subjects are tested in standardized conditions (García-Loor et al. 2025). Nevertheless, additional measures of territory defense investment, such as song rate or time spent near the nest, may provide a more nuanced measure of the time and energy costs associated with male territory defense (Ord 2021).

During the incubation and chick feeding stages, we found no relationship between a male's territory defense behavior and his food delivery rate. This suggests that responding more aggressively toward territory intruders does not necessarily detract from parental care in Darwin's finches. These findings are inconsistent with previous work in house wrens (*Troglodytes aedon*; Barnett et al. 2012), blue tits (Mutzel et al. 2013), and white-rumped swallows (*Tachycineta leucorrhoa*; Wischhoff et al. 2018), all of which reported negative correlations between territory defense and provisioning rate. Of course, such tradeoffs are not ubiquitous in the literature and may be species-specific: previous studies reported no correlation between these variables in song sparrows (Lane et al. 2023) or collared flycatchers (*Ficedula albicollis*; Szász et al. 2019b) and a positive correlation in female house wrens (Krieg and Getty 2020). In Darwin's finches, the predicted tradeoff between territory defense and parental care may manifest in ways that were not measured in this study. Notably, we did not identify food items brought to the nest, so it is possible that variation in parental care arises through the size rather than frequency of food deliveries (Grieco 2002a). This is a plausible source of variation in Darwin's finches, which have comparatively low rates of nest visitation (0 to 9 visits/h; Kleindorfer et al. 2021) and so may dedicate considerable time between visits to searching for and extracting food items. This would be especially true for tree finches, which specialize in the subsurface extraction of invertebrate prey from woody substrates (Kleindorfer et al. 2022). It remains to be tested whether aggressive males compensate for their increased investment in territory defense by reducing their “critical prey value” during foraging (sensu Grieco 2002a, 2002b) and so delivering smaller or less nutritious items to the nest.

The challenge hypothesis provides a mechanistic explanation for the tradeoff between territory defense and parental care. Specifically, it predicts that males increase their testosterone secretion in response to territorial interactions, which consequently suppresses their parental care behavior (Wingfield et al. 1990, 2020). Predictions of the challenge hypothesis are well supported in the literature but not across all taxa (Lynn 2008; Goymann et al. 2019; Moore et al. 2020). In particular, the association between testosterone and parental care tradeoff likely depends on each species' mating system (Stiver and Alonzo 2009). In biparental species where paternal care is essential, selection may favor males whose parental behavior is less sensitive to increases in testosterone, thereby weakening the tradeoff between territory defense and parental care (Lynn 2008). Darwin's finches could certainly qualify as a mating system with essential paternal care, given that males deliver food to the female during incubation and share nestling provisioning duties.

Female Darwin's finches that were paired with aggressive males spent less time incubating their eggs. This suggests that there may be a weak within-pair tradeoff between territory defense and parental care—however, the mechanisms mediating this relationship are unclear, given that male-to-female food provisioning did not differ between aggressive and nonaggressive males. Differences in female time on the nest could have consequences for thermal insulation of offspring (Martin et al. 2007), egg predation (Rastogi et al. 2006), or frequency of oviposition by the parasitic avian vampire fly (Kleindorfer et al. 2021). Although female Darwin's finches are the sole incubators and often receive food from their partners, they also regularly leave the nest to forage for themselves or chase away intruders (personal observations by authors). If Darwin's finches tend to mate assortatively for aggressiveness, as has been reported for other songbirds (eg in eastern bluebirds, *Sialia sialis*; Harris and Siefferman 2014; Burtka and Grindstaff 2015), then the partners of aggressive males may themselves also spend more time off the nest engaging in territory defense.

Male territory defense during the incubation period did not predict the likelihood of hatching success, which implies that both aggressive and nonaggressive strategies achieve equal fitness during this stage of the breeding cycle. Because our study is based on a single season of data, it is possible that the prevailing environmental conditions weakened any potential tradeoffs between behavior and fitness (Thys et al. 2021). This study was conducted on Floreana Island in early 2024, during abundant El Niño conditions that are generally associated with high food availability and high reproductive productivity in Darwin's finches (Gibbs and Grant 1987; Grant et al. 2000). This period also followed shortly after an island-wide mammal eradication campaign in late 2023, which not only removed most of the island's nest predators but also greatly reduced Darwin's finch populations due to nontarget poisoning (personal observations by authors). For the island’s surviving finches, conditions may have been so optimal—with high food availability, low conspecific densities, and low rates of egg predation—that any tradeoffs associated with territory defense were smaller than usual. However, longer-term monitoring is needed to explore how this relationship fluctuates across years.

In this study, we found only weak evidence for a tradeoff between territory defense and parental care in Darwin's finches. Although individual differences in male territory defense behavior were highly repeatable, being more aggressive did not detract from males' nest provisioning effort. While female finches with aggressive partners spent less time incubating their eggs, the behavioral mechanisms mediating this relationship remain uncertain.

## Data Availability

Analyses reported in this article can be reproduced using the data and R code provided by Katsis et al. (2025).
